# Anthropomorphizing with Critical Reflexivity: The Danger and Potential of Anthropomorphizing in Equine-Facilitated Learning and Psychotherapy

**DOI:** 10.3390/ani15040605

**Published:** 2025-02-19

**Authors:** Kelsey Dayle John, Aviva L. Vincent, Leanne O. Nieforth, Jamie Schafroth

**Affiliations:** 1 Ethnic Studies, College of Arts & Sciences, University of Colorado Boulder, Boulder, CO 80309, USA; 2 School of Social Work, College of Health, Cleveland State University, Cleveland, OH 44115, USA; a.l.vincent@csuohio.edu; 3 Comparative Pathobiology, Center for the Human-Animal Bond, College of Veterinary Medicine, Purdue University, West Lafayette, IN 47907, USA; lniefort@purdue.edu; 4 Anthropology, College of Social and Behavioral Sciences, University of Arizona, Tucson, AZ 85721, USA; jschafroth@arizona.edu

**Keywords:** anthropomorphism, equine-facilitated learning, equine-facilitated psychotherapy, critical reflexivity, horses, welfare

## Abstract

This article used an intersectional feminist analytic framework to theorize the role of anthropomorphizing human–equine interactions in the HERD Institute’s modality for practitioners of Equine-facilitated learning and equine-facilitated psychotherapy (EFL/P). EFL/P is a traditionally human-centered activity which leverages anthropomorphizing equine behavior and emotions to better understand human selves and dynamics by assigning human emotions to equids and human–equine interactions. Intersectional feminist theories were used to interpret 11 in-depth interviews with EFL/P practitioners. The data analysis identified anthropomorphizing behaviors which were used as the foundation for the creation of two vignettes. The vignettes present different ethical dilemmas of anthropomorphizing that may impact equine welfare in EFL/P explicitly discussed by interlocutors. One vignette examines interpreting and assigning human injustices within equine behavior (e.g., resource guarding) and the second engages with human interpretations of equine choice versus consent in an EFL/P session. The vignettes demonstrate how anthropomorphizing may be used to develop an understanding of human dynamics or equine welfare. The researchers suggest that the use of critical reflexivity when anthropomorphizing may increase attention toward equine welfare in EFL/P settings. As such, it may be beneficial to consider the development and refinement of EFL/P training/certification and continuing education.

## Simple Summary

Equine-facilitated learning and psychotherapy services are traditionally a human-centered activity that leverages anthropomorphizing for the benefit of humans and at the expense of equine welfare. Feminist theories can be used to critically analyze human use of anthropomorphizing in these settings. Using interview data from the HERD Institute, the researchers constructed two vignettes to illustrate how critical reflexivity may help harness anthropomorphizing for the benefit of equine welfare and human development.

## 1. Introduction

In human–equine interactions, many humans, including our research team, have wondered if we are correctly interpreting a human–equine interaction, or if we are fairly or unfairly imparting our own human beliefs, thoughts, and feelings onto the equids—in what is known as anthropomorphizing [1]. For those who have training in equine behavior, this internal dialog can become even more confusing. Drawing from the definitions offered in the recent literature [2], this paper used the word “facilitated” when speaking about equine-facilitated learning and/or psychotherapy rather than “assisted” to honor the language of the provider we partnered with. We are also aware that Nina Ekholm Fry [3] had also offered the following phrasing “horses who participate in human services” which honors the human-centered nature of such services. In line with Fry’s offering, conversations about EFL/P have become more focused on equine welfare in addition to the wellbeing of humans [3]. A critical reflexive interpretation drawing on feminist animal studies would argue that equine welfare is directly connected to the marginalization of humans and that there are shared structures of management that affect equids and marginalized humas [4,5,6,7,8].

In human–animal interactions as a whole, scholars have suggested that critical anthropomorphism might be a way to make sense of these complex human–animal interactions without being reductive [4,5,9]. In this paper, we used our expertise as social science researchers, who focus on human–equine interactions, to provide a discussion about the “critical” aspect of the critical anthropomorphism. In doing so, we borrow from a long-established field of intersectional and feminist reflexivity to develop a *critical reflexivity* that can be used by practitioners of EFL/P.

In the current literature, the least developed area of reflexivity is practitioner and client awareness of human power dynamics and how those dynamics overlap and intersect with animal’s lived realities. There is very little information about whether practitioners also have training, education, and/or personal experience with the structural areas of human power dynamics, inequality, and social dynamics, especially in EFL/EAL [10] due to the de-professionalization of EFL/EAL.

To illustrate anthropomorphic themes and how practitioners may respond, the authors created two vignettes, using interview data with EFL/P practitioners of the Human Equine Relational Development (HERD) Institute [11]. The HERD Institute provides specialized training for EFL/P practitioners. All practitioners of the HERD Institute are required to sign a commitment of belonging. On their website mission statement, the HERD institute writes, “we know that in order to create such a space [inclusive], we must actively and consistently do the work around dismantling systems that continue to oppress those who have historically been underrepresented” [11], illustrating a commitment demystifying and dismantling systemic barriers and injustices within EFL/P. Following each vignette is a discussion about the practitioner’s use of *critical reflexivity* and choice points that may arise on how to respond to anthropomorphizing behaviors in EFL/P settings.

It is important to note that this publication is one of three in a series. Each of the three publications serves to build on a specific element of introducing and building intersectional and feminist theory for areas in human–animal interaction broadly referring to “any manner of relationship or behavior between people and animal(s)” [12] and human–equine interaction as a whole. Our critical reflexive approach drawing on feminist animal studies engages with the assumption that equine welfare is directly connected to the marginalization of humans and that there are shared structures of management that affect equids and marginalized humans [9,13,14,15,16].

### 1.1. Phenomenological Approach to EFL/P

Within the HERD EFL/P models, the intention of practitioners is to use a phenomenological approach and stay embodied to experience the sharing of space with equids—or a human–equine interaction; referred to as the “here and now” [7]. The practice of embodiment with equids (or a phenomenological approach) trains practitioners to be present in “the here and now”, to experience what the HERD institute calls an “I and thou” relational moment where a person recognizes another (human or equid) [8].

A phenomenological approach to EFL/P welcomes the attribution of human emotions, experiences, and dynamics onto equids and their behavior (if this is what the client is experiencing) and the practitioner’s responsibility is to guide the client through an exploration of meaning-making. The HERD Institute has voiced a commitment to addressing such experiences through an understanding of human structural forms of inequality.

### 1.2. Critical Anthropomorphizing in EFL/P

One study on human–equine interaction found that humans use anthropomorphic language and analysis to understand the connection they perceive between the equid and themselves [17]. In broader conversations, scholars have introduced critical anthropomorphism as one option for working mindfully with anthropomorphizing; this process involves the commitment to use both “scientific and empathetic approaches to study animals” [5] (p. 709). To contextualize anthropomorphizing, Randall Lockwood [1] traced the practice within animal policy arguing that, historically, assigning emotion, sentience, and complex thinking to animals was “dismissed as sentimental or “anthropomorphic”’ [1] (p. 103) to delegitimize welfare concerns for animals. He wrote,

for most of the last century, the prevailing view had been that the use of anthropomorphism in describing or explaining non-human behavior undermined the objective, rational basis of animal studies, in an era when many scholars in comparative psychology were striving to put the field on an equal footing to physics” [1] (p. 96).

The legitimacy of anthropomorphism seems to hinge on whether it upholds notions of scientific objectivity or not. An intersectional feminist analytic framework may be a useful tool to engage with a paradigmatic analysis of human–equine interactions within scientific analysis and EFL/P, since feminist scholars have long critiqued the notion of pure scientific objectivity [18]. In a public thought piece, Michelle Quirk [19] summarized the conversation on anthropomorphizing and suggested that humans use what she called “mindful anthropomorphizing”. Our use of *critical reflexivity* differs slightly from both existent frameworks, although it is related to “mindful and critical anthropomorphizing” within the context of human–equine interactions. *Critical reflexivity* draws on critical frameworks offered from feminist scholarship traditions [20,21] to better understand and navigate macro–level human influences within EFL/F microcosms.

### 1.3. Critical Reflexivity

Reflexivity is a popular term in feminist studies and marks one’s practice of engaging with questions about one’s own positionality, or position within society and its different power structures, as well as how that positionality may affect their thinking and interpretation of relationships. In the current work, we consider how *critical reflexivity* not only impacts research practices, but how it may impact the decisions that EFL/P practitioners make related to anthropomorphic interpretations of human–equine interactions. Reflexivity is different than reflection in that the practice asks a person to intentionally reflect on the structural components of their position in society in relationship to their meaning-making process. In this case, meaning-making happens when a practitioner facilitates a learning or therapeutic experience with equids.

The HERD Institute’s phenomenological approach to EFL/P compliments *critical reflexivity* in that it expects practitioners and participants to make sense of their relations through an acknowledgment and inquiry about themselves and their own embodied reactions, perceptions, and interpretations. *Critical reflexivity* can be brought in for processing these experiences, which may happen in a session or post-session at a later point in time. It can also be used to help practitioners hold ongoing tensions, contradictions, and nuances in their work.

Hesse-Biber and Piatelli [22] explained how the practice of feminist reflexivity is grounded in feminist critiques of science and epistemology. They wrote, “a feminist epistemology questions the proposition that the social world is one fixed reality that is external to individual consciousness and suggests that this world is socially constructed, consisting of multiple perspectives and realities” [22] (p. 7). In both research and education, scholars have used *critical reflexivity* as a practice to acknowledge one’s positionality and relationship to structures of power as well as an active and embodied understanding of how those relations might play out in interpersonal relationships and meaning-making [23].

In teaching and education, Feucht and colleagues [24] wrote that reflexivity is different than reflection (or an individual thinking about interactions and events) because it is an “informed and intentional internal dialogue [which] leads to changes in educational practices, expectations, and beliefs” [24] (p. 234). Intersectional feminist scholarship provides a shift in critical thinking that zooms out from individual interactions and instead provides the infrastructure to think about how interpersonal interactions are situated within broader paradigms related to power, oppression, and systemic inequality regarding settler colonialism, white supremacy, heteropatriarchy, and classism [25]. Black feminist scholars have introduced the first and most well-known analytical framework in the field called intersectional theory to show how there is no singularly operating oppression system, but that they are all intertwined in complex ways [20,21,26]. Furthermore, there is a shared nexus of oppression among and between humans and animals [9,13,27,28,29,30]. We addressed these connections in more depth in one of three publications from this research [6].

To our knowledge, intersectional feminist theories are only occasionally used to think about human–equine interactions and not applied to EFL/P [6]. Some known considerations regarding human–equine relationships include Gala Argent and Andrea Vaught’s [9] edited volume which explained that “the narratives which we hold as true about the capabilities of horses—both academic and popular—legitimize, allow and delimit where the horse is situated within human intellectual, cultural, economic, and political spheres. Moreover, these beliefs both reflect and influence how we perceive (and study) our relationships with horses, the work we ask of them, and the ways in which they are treated as they carry out that work” [9] (p. 2). The authors suggest the use of feminist ethics to theorize human–equine relations because there are “explicit and implicit hierarchies that cross species boundaries” [9] (p. 11) that humans need to pay careful attention to.

### 1.4. Equine Welfare

In addition to power, oppression, and systemic inequality among human structures, our construction of *critical reflexivity* contends with three important areas of equine welfare within EFL/P that may intersect with anthropomorphic logic when interpreting and responding to human–equine interactions: responsibilities in EFP/L, dominance theory, and navigating consent verses choice. In addition to these three areas, *critical reflexivity* also helps practitioners address human intersectional experiences (for example class, race, or gender) that might be reflected in human–equine interaction (we discuss the gap in this training and awareness in Vincent and colleagues) [10].

Nina Ekholm Fry [3] wrote that it is important for practitioners of EFL/P to educate themselves about equine welfare. Oftentimes welfare is assumed in human services; however, she noted that, “the context of human services does not automatically offer protection against the lack of recognition of the mitigation of equine welfare issues” [3] (p. 47). She cautions that many people who work with equids might ignore issues of welfare for four reasons: (1) Not being able to identify the problem, (2) Misinterpreting equine communication, (3) Following bad advice from those in charge, and 4. Desensitization to consistently normalized poor welfare. The basic needs of equids are summarized as: “free movement, forage, social housing, and choice in their everyday lives” [3] (p. 49), meaning that these principles should apply to the everyday lives of equids who work in human services and not just during sessions. Fry offers five responsibilities for practitioners while facilitating EFL/p sessions with respect toward equine welfare: “1. Monitor interactions and track affective states, 2. Assess and address handling and touch, 3. Communicate accurately about the horse and the interaction, 4. Assess stress, and 5. Promote positive states” [3] (p. 51–52).

Scholars have suggested a reflective approach to infer when anthropomorphizing equids is useful or harmful to the equids’ welfare [31]. Accounting for equine welfare also means accounting for the equid’s history, just as a practitioner would do for a participant. All equids enter a session with past experiences and preferences. While practitioners trained in phenomenology focus on the equid’s existence in the “here and now”, it would be a detrimental omission to ignore the equid’s full context, including their history of experience, temperament, and health challenges [32]. By accounting for the species and individual needs, EFL/P practitioners can make decisions about appropriate programming, engagement, and workload.

### 1.5. Dominance Theory

The human-created idea of a social hierarchy reinforced by equine-on-equine dominance is a persistent idea within human understandings of, and interactions with, equids [33]. For the most part, the persistence of dominance hierarchies has benefited humans as a justification of controlling equids within interactions [34]. There is also evidence that such hierarchies are related to areas of power, oppression, and systemic inequality mentioned in the earlier section [35]. However, through the observation of wild equids, scientists have found that rigid dominance hierarchies do not exist in herds, providing evidence that relationships are more fluid and non-hierarchical [14,33]. Rather, equine social relationships are organized around three distinct spatial principles that keep wild equids safe: “stay together, synchronize, and respect other’s space” [34] (p. 25). Furthermore, Rees [34] explained how competition over resources (i.e., food, space, shelter) exists only insofar as there are human-created structures that promote scarcity and create increased competition over restricted access to resources. Thus, food management practices in domestic settings often cause increased aggression among equids, but aggression or hierarchy is not their homeostasis.

### 1.6. Consent vs. Choice

Based on the existing literature and our application of *critical reflexivity*, choice is when an equid has choices within a session to perform one thing or another. Consent is when an equid has the option to opt in or out of the entire interaction/session. In a piece authored by the HERD Institute founder, Veronica Lac [36] explained the difference between compliance versus consent where compliance means the participants are doing something to the equid and ignoring signs of engagement or discomfort. Conversely, consent occurs when the participant asks the equid to engage with them and allows time and space for the equid to step into participation or to choose to disengage. Consent allows for the participant’s acceptance of the equid’s choice. By this standard, the practitioner must be aware of the “equine[‘s] ethology, behavior, stress, and pain signals” [36] (p. 109) to make distinctions between compliance and consent and then follow through with important decisions about the interactions in session. This aligns with the researcher’s definition of these constructs. Lac [36] further explains how consent and choice relate to equine welfare: “our methodology is based on a foundation of social justice, diversity, equity, and inclusion, and awareness of power, oppression, and cultural sensitivity issues are applied to both equids and humans, as they often emerge in the process in parallel” [36] (p. 104). In the chapter, Lac explained how the client population and their experiences with force, dominance, and lack of consent impact how the practitioner makes choices within a session.

Assessing equine consent to participate in human services is still an ongoing conversation and is an area that needs more research. It has been noted that a trauma-informed approach is useful for assessing consent within human–equine interactions [3,14]. However, Seery and Wells [31] found in their research with EFL/P providers that “the issue of horse consent and advocacy” was ranked lower than other areas of concern which they suggest needs additional research.

## 2. Materials and Methods

### 2.1. Data Collection

The research team conducted 11 in-depth, semi-structured interviews [37] with adult EFL/P-certified practitioners and practitioners working on their certification with the HERD Institute. Interviews were conducted between May 2023 and May 2024. This study was approved by the University of Arizona Institutional Review Board (Reference number 00002719). Participants were recruited via social media and the HERD listserv. Interviews were conducted via Zoom by one of the four research team members. No demographic information about participants was collected because the HERD trainee and graduate pool was a small community; any reported demographic information would likely compromise a participant’s privacy within the study.

The semi-structured interviews were designed to explore understandings of diversity, equity, and inclusion within EFL/P with seven guiding questions; the interviewer would add additional follow-up and probing questions [38] based on the information shared by participants. In this paper, we focused on themes that arose around interview question *3:* “Can you describe a moment where you became aware of the parallels between human and horse social interaction and/or instances of power?” This question was designed to capture if/when participants and practitioners were making connections between human social systems and equine interactions they observed in their training/practice.

### 2.2. Thematic and Content Analyses

Zoom interviews, lasting approximately ninety minutes, were recorded, transcribed, checked for accuracy, and hand-coded using thematic and inductive coding [23,39] by each member of the four-person research team. After individual coding, the team met to compare and develop a jointly coded memo by comparing our individual codes and using dialogue to summarize and note the major themes [40,41,42]. The research team dialog was a part of our own researcher reflexivity [4,43,44]. It was in the creation of a joint memo that the research team spoke at length about themes of anthropomorphism observed in the data. This jointly coded memo was then shared and member-checked with the participant in a follow-up conversation. Follow-up conversations were not recorded but notes were taken, and these conversations were used as a part of the analytic process.

In the interviews, the theme of anthropomorphism arose quite often, implicitly and explicitly, and sparked a lively dialogic analysis among the research team regarding these moments within EFL/P. Rather than just presenting anthropomorphism themes (i.e., raw data) from our interviews, our team decided to use the data to construct two vignettes [45] to capture the complexity of anthropomorphizing in EFL/P and how practitioners might respond.

### 2.3. Vignette Development

The vignettes illustrate how instances of anthropomorphizing show up in the EFL/P sessions and narrate how the practitioner might use *critical reflexivity* to interpret and eventually respond within and beyond the setting for the betterment of equine welfare. The goal was to synthesize the data into two vignettes that captured the complexity of human–equine interactions and the case of anthropomorphizing within service settings and map the direct impact on equine welfare in EFL/P. Researchers have “used vignettes to explore the ways in which actors perceive and respond to social situations, especially ‘sensitive’ social situations that may be practically or ethically difficult to observe first-hand” [45] (p. 976).

Construction of vignettes started with reviewing the jointly coded research memos for anthropomorphizing themes. Then, the researchers proposed ideas for vignettes that brought multiple themes together in one story. After drafting vignettes, the authors checked validity and context by revisiting the interview transcripts, individually coded memos, and follow-up conversations. The team also asked two non-HERD EFL practitioners to review the vignettes for plausibility. Relevant to the reflexive process, the researchers also drew on their backgrounds as EFL/P practitioners to contribute to a probable vignette based on lived experiences.

## 3. Results

Below are the five areas of anthropomorphic behaviors identified within the interview data. In the vignettes, we noted in parenthesis where the following themes have influenced the construction of the vignette.

1. *Critical anthropomorphism:* Multiple practitioners referenced their engagement with equine scientific literature to better understand interactions. One practitioner spoke in depth about their intentional and careful work to not anthropomorphize equids and equine behavior offering a critical framework through which they referenced scientific literature to help with these choices.
2. *Interpreting vs. observing*: Practitioners’ reflection demonstrated their process of dealing with anthropomorphic assumptions. For example, one practitioner warned that without critical reflection you stop noticing and you start interpreting, showing that they knew that human interpretations of behavior could be problematic and predetermined.
3. *Projecting human experiences on horses*: Practitioners expressed awareness that anthropomorphizing could result in humans seeing the horse as a mirror of their own emotional state or practicing transference which could lead to poor welfare decisions based on the incorrect interpretation of the horse’s behavior.
4. *Communicating directly with horses*: Practitioners shared experiences of communicating with horses, experiences with horses talking directly to them, and experiences interpreting horse behavior and interactions.
5. *Social and systemic power systems*: Practitioners made macro-level connections between horses, typically mustangs, and their connection to systemic inequality shared by Black, Indigenous, and People of Color.

While thematic responses were fluid and individual, the conversation often flowed and circled back around to the third theme. Though not directly a theme of anthropomorphism, it was a theme that was common in conversation (and relevant in our vignettes). Practitioners’ beliefs about equids and anthropomorphizing spanned the particular space of EFL/P and was apparent in all areas they spoke about including training, equine science, competition, cultural perceptions, equine ownership, and EFL/P work/training.

### 3.1. Vignette #1 Structural Inequity and Food Insecurity Among Equids and Humans

In an EFL/P session, three equids are at liberty with three hay bags placed in an arena for consumption. As the session starts and the equids are let into the arena, where they remain at liberty/loose, one equid starts eating from a hay bag as a second equid approaches the same hay bag. The first equid eating from the hay bag pins his ears back and chases the second equid away the hay bag he is eating from. The client then responds saying, “that horse is bullying the other horse; they’re not sharing their lunch with the other horse” (Theme 2; Theme 5).

This participant is a member of a marginalized group, a single mother, and has shared in other sessions about their struggle to afford groceries for their family. The client expresses that they are uncomfortable with the equine interaction because it seems unequal. The practitioner internally perceives that this participant has a history of food insecurity, and the situation might be bringing up feelings of inequitable access to resources (Theme 3). The EFP practitioner asks, “what is coming up for you?” and the client responds, “I interpret this interaction like those in power, people with more money or white people guard and keep resources for themselves and their families, maybe excluding people of color or poor people” (Theme 5).

The EFP practitioner has a significant amount of equine behavior training and is aware that resource-guarding behavior among equids is due to human-imposed feeding and management systems throughout the equid’s life and not just the session (Theme 2). In this instance, the practitioner knows that the client is having an experience where they are anthropomorphizing based on their experience of human societal inequities and using this framework to interpret the equid’s interactions due to the management systems created by humans (Theme 5). The practitioner wants to use their equine behavioral knowledge as well as their systemic knowledge of human food insecurity to make a decision that will ensure the most comfortable environment for both equids and humans. They are also aware that the stressful situation regarding food access may not necessarily be because of the set-up for their session, as the equids have been provided with free choice of hay and body movement during this session.

The practitioner also knows that choices are contingent upon the facility, the clients, the structure of the program (i.e., EFL vs. EFP), and the duration of the session (i.e., number of changes that can be made (or not) in this setting). The practitioner postulates several scenarios before they proceed with questions for their client.

### 3.2. Potential Questions the Practitioner Might Ask the Client in EFP Framework

What story are you telling yourself about what is happening here?
Do you have to decide right now?
Where are you feeling [sensation: tightness, movement, release…] in your body?
How would you like to manage these feelings?
What would you like to do in this moment?
What choices can you make?
Are you safe sitting in stillness?
What can you control here?

### 3.3. Choice Points for the Practitioner

The practitioner might share that the equid’s behavior is contingent upon the its overall management system but knows they personally cannot change that management and neither can the client, so it might engender feelings of hopelessness. If the client and practitioner follow the idea that this equine-on-equine interaction is mirroring human social oppression (i.e., racism or classism and the resulting resource control) without noting that resource-guarding behavior in this setting is due to macro-level management concerns, then the responsibility for a human dynamic falls on the equid who is doing the guarding. So, they may interpret this as: “that horse is acting white/wealthy; they are acting dominant” (Theme 5).

If the practitioner makes that anthropomorphic interpretation, then the client may make the choice of tying up the equid, separating them from the hay, or removing them from the arena—all of which could negatively affect the equid’s welfare and ignore the larger structural inequalities (on the equid and human’s behalf) that are contributing to the experienced moment. The practitioner is responsible for considering the action that can be taken in the moment, if any action is taken. The client may request to move one equid away by haltering or using their body. The client may even want to move the equid to another pasture. If the client chooses to halter the equid, they have choices of holding the equid, taking them to a different hay bag, removing them from the arena away from herd-mates, or tying them to a structure.

The practitioner also must be aware that when clients make suggestions, such as to move equids, they do not have to follow through with that action for the suggestion to be effective. The practitioner has the responsibility to decide whether to suggest or accept a change or to discuss the situation at hand. Each choice of action or inaction has a different implication for the equine’s welfare and could have a different implication for the client’s processing of the session.

### 3.4. Use of Critical Reflexivity

The intrapersonal interaction comes back to the central question of “what is the role of the horse?” in the EFL/P setting. Technically, the participant(s) needs to benefit educationally or therapeutically from the interaction with the equids more/differently than they would in an environment without the equids present.

Rees wrote, “fighting is not a matter of “working out the hierarchy” but a result of poor management and welfare, as perceptive horse-keepers realize” [34] (p. 29). Practitioners then must pay attention to the management situations (using *critical reflexivity*), with a greater systemic focus on the entanglements between human services and equine welfare. We, as researchers and practitioners, know it might not be “reasonable to have horses’ part of human services who are not able to move freely during the day or night, who are fasting for longer than 3–4 h at time, and who are not housed together with other horses due to lack of space” [3] (p. 21). In this moment, the client, the practitioner, and the facility’s participation in the equine’s management are perpetuating the structures resulting in the observed behavior of the client and the equids. The practitioner must sit with this conflict and still make the best decisions in their session.

### 3.5. Vignette #2: Consent vs. Choice

In an EFL group session, two clients are working on communication skills. The practitioner has decided to incorporate picking the equids’ hooves into the session. Two equids are at liberty in an arena. The practitioner directs clients to approach an equid, pick up their hoof, and pick out their hoof using a hoof pick. The practitioner wants to be aware of consent and choice so the first intention is that the equid can consent to sharing its space with the human because they are at liberty. The secondary intention is that the equid can consent, if they want, to lift their foot and allow the hoof to be picked, since they are not haltered.

In the session, the two clients respond differently to the situation. One client wants to “take charge” and pick up the equid’s hoof with minimal attention to the dynamic between themselves and the equid. While approaching the equid, they mention an expectation formed by their competitive background that equids “should keep their feet up for as long as the human needs them to” and states that this is for the equid’s ability to receive farrier work and for the safety of human and equid (Theme 1). As the person approaches, the equid stands still, with ears perked head at the level of their withers, and four hooves flat to the ground. As the client aims to lift the front hoof, the equid shifts their shoulders away, moving their body into a “U” shape away from the client.

The second client is more stand-offish with the equids and is unclear on how to ask the equid for their hoof. They approach the equid with confidence but then lose track of the interaction. The equid stands still with three hooves planted and the rear hoof resting on the toe. The equid has ears moving forward and to the side, head at the level of their withers, eyes glazed over. As the client tries to ask for the hoof, the equid yawns, but does not move their hooves. As the participant stands with the equid, the practitioner notices that the client looks to be almost in tears. The participant says aloud that they can hear the equid saying: “I don’t want to pick up my foot” (Theme 4).

In both instances, the equid is making a choice not to consent. It is likely that both clients are anthropomorphizing, though in different ways: First, one person thinks the equid should be able to abide by human timelines, constructs, and expectations for equine welfare. Second, the other person claims the horse is talking to them directly, expressing their desire not to pick up their hoof (Theme 4), but it is likely an impartation of the client’s lack of confidence or discomfort with picking up the hoof (Theme 3).

### 3.6. Potential Questions the Practitioner Might Ask the Client

What does it mean to “be in charge”?
What do you want to control right now?
If equids need to pick their hooves up for safety, are you saying that they are unsafe right now?
What does it mean to be safe?
Are you asking your equid to lift this hoof?
How are you asking your equid to lift their hoof?
What is the equid asking or telling you?

### 3.7. Choice Points for Practitioner

Picking the hooves is often presented as a confidence-building activity. The outcome is for the clients to converse with equids while also embodying confidence in asking the equid to engage with them. Since the first client defines equine welfare as the intent of picking up the hooves, the practitioner has the choice to be explicit or implicit about how equine welfare shows up in this moment and in the broader management of equids. The modality of EFL/P may dictate the scope of practice and whether a practitioner chooses to navigate a conversation about feelings and emotions or focuses on learning with equids. Even when the conversation or behavior feels like it is no longer about the equid, it will likely turn back to the equid; the practitioner’s patience is crucial.

The practitioner can inform the client that for the equid to understand what they are asking, they need to present a specific and clear cue (and demonstrate what that may look like). For the equid in the vignette, it is the pressure on the inside of their hock that releases the moment they shift their weight. Helping the client pick up the equid’s hoof and working on their embodiment of confidence and authority might be useful in building interpersonal skills.

However, shifting into action ignores the conversation surrounding the definition of consent. The other client may view this as ignoring the equid’s “no.” The practitioner can confirm that the client has heard the equid talk to them and what that means to them by clarifying that the equid “does not want their foot picked up”. However, the practitioner knows, from their training, that asking equids to move their bodies is best done through a clear application of learning theory [46] which this client has failed to do with their body language and the use of a reinforcement. They also recognize the yawn as a stress signal. Although these participants are not “training the equid” for hoof picking, the practitioner is aware that their activity is reinforcing behaviors through cues that are either clear or unclear, which results in the equine’s continual conditioning having an impact on their welfare.

### 3.8. Practitioner’s Use of Critical Reflexivity

The fundamental questions for the practitioner to consider are: (1) What is the point of picking up an equid’s hoof in this session? (2) Is it important that the equid picks up their hoof or is the goal to have a conversation around the equid’s response? And how does this return to the intention of this EFL session designed around communication and equine consent/choice?

The practitioner can use *critical reflexivity* to explore the use of spoken human-centric language (even in respect of the equine’s welfare), and how questions are asked to equids. Exploring language and intent of language between equids and humans inherently includes engaging with anthropomorphism as well as power. Humans use language, vocal intonation, body positioning, and touch. Is the person narrating their own meaning from their own internal experiences (an EFP interpretation)? If so, we wonder how this affects the welfare of the equid because the focus is on the human’s experience and ability to hear from and interpret the equine’s behavior. The session is not about understanding the best practices for training—which studies have shown are a major consideration of welfare in EAS [47]. However, the human’s interpretation is privileged and not being filtered through that understanding of equine training. When a human claims an equid talks to them, they are applying an interpretation and must be mindful to keep observing while filtering that interpretation through their knowledge of scientific information. A good interpretation would be filtered through scientific interpretation, in the moment of observation, as well as personal experience [4] (p. 12–13). Will the practitioner step in if the client hears from an equid directly but ignores a simple explanation about the situation—that the equid simply does not understand the cue being given to them from this human? The practitioner is responsible for conceptualizing choice and consent for both the equids and humans.

## 4. Discussion

### 4.1. Practitioner’s Use of Critical Reflexivity

It is important to note that the *critical reflexivity* does not end with either session, but that the practitioner (and perhaps the client) will likely continue to sit with the larger questions of food insecurity, consent/choice, equine welfare, and the equid’s experiences during sessions. They may begin to make parallels between the management and control of equids and the management and control of marginalized humans (as Black and Indigenous scholars already have), or the paths of communication offered for human–equine interaction.

On a macro level, we ask: is the management of equids who engage in resource-guarding during a session necessary for this client to process their emotions around food insecurity? Rees [34] argued that nearly all management strategies humans consider “normative” lead to aggression (i.e., limited space, small paddocks, unnatural herd groupings, social education, limited access to food, and pain from mounted work). Human food insecurity is not always due to direct interpersonal domination but can be understood as systemically controlled based on one’s income, geographic location, race/ethnicity, historical oppression and trauma, legacies of colonialism and enslavement, and other interpersonal and systemic factors. A practitioner using *critical reflexivity* will be aware of and able to hold all these dynamics at once during and after a session.

In the first vignette, anthropomorphizing could mean the session was centered on the client’s projections of their food insecurity onto the equid, with the idea that the equid is “mirroring” the actions of the human in the session or that equids and humans share power dynamics across species. Both interpretations do not consider the equids’ management and why resource-guarding may occur between domesticated equids in a domestic setting. Viewing the interaction through an EFL/P human-centric lens could lead to a situation (if consistently replicated) that impacts equine welfare and, in the case of resource guarding and management, it already has. It also elides a broader discussion about how humans control all aspects of equine life for EFL/P equids inside and outside sessions.

Using *critical reflexivity* to guide the inquiry process offers choice points as well as long-term considerations for practitioners of EFL/P. The practitioners can use *critical reflexivity* to potentially shift the conversation away from an interpersonal construct and towards a structural investigation of areas like food insecurity, which might help participants focus on macro-level structures among humans in EFL/P. This is an opportunity to form connections in the therapeutic process and has potential to help engage clients in a process of changing the culture of the EFL/P industry—which scientists suggest is needed [3]. In this instance, talking about equine behavior or welfare may aid the learning or therapeutic process by giving clients a different opportunity to connect with equids because they think about a shared presence in different and overlapping structures. However, this can only be achieved when a practitioner is aware of how those structures might be shared and differ, and not just perceived as shared experiences that negate either the equid or marginalized human’s lived reality. This is the very practice of *critical reflexivity,* by understanding that the equid and human might both experience food insecurity and that is both similar and different at the same time. To be effective, an in-depth knowledge of human power structures and their effects on human–equine interactions, as well as the ability to sit with differences, similarities, and contractions, is essential.

In vignette number two, the practitioner can use *critical reflexivity* to decide how to talk about and enact consent with attention toward human and equine consent. In the human context, consent is most discussed as an interpersonal experience between two humans, often for sexual engagement but it need not always be understood in this context, as someone may consent (or not) to join in on a conversation. Thus, talking about consent brings up different feelings or thoughts from different people, so we advise that practitioners proceed with care. More importantly, discussing lack of consent can trigger negative connotations of equine behavior. However, calling out experiences of non-consent can be one of the most formative experiences for relational learning. If the participant needs to express that the equid is opting out or claims the horse is talking to them, saying they are opting out as a way to express their own boundary or lack of consent, the practitioner might choose to honor this.

With *critical reflexivity* the practitioners must also think long-term and ask: given the equid’s history, does the equid suffer from stress related to their work? For example, if a client perceives that they are not receiving consent from the equid to pick up the hoof and chooses to continue their pursuit in the activity, they may be engaging in domineering or controlling behavior. At the same time, ignoring the client’s discomfort with the equid not consenting could seriously impact a client with a history of sexual assault. While we know that much of human–equine interaction is constructed around the expectation that equids will be controlled, managed, and dominated by humans, consent as defined herein, is also a human-informed construct that is impossible to measure objectively. Thus, the practitioner must consider if it is more important to engage the client in a conversation about awareness of consent from what we know based on equine behavior research [48] and if it is an equine welfare issue to engage in an activity without explicit consent. In the process of exploring anthropomorphism and equine welfare, there is a unique question for the practitioner: although the equid(s) have choice in the session, have they consented to doing this work in general and how can that be confirmed? We may suggest more work to normalize the idea that human–equine interactions within the EFL/P setting might not involve touch or could involve little to no physical interaction at all.

### 4.2. When Behavior Meets Relational Experiences Rooted in Anthropomorphism

Seery and Wells [31] have written that the 41.5% of practitioners in Equine-Assisted Services, inclusive of EFL/P, have little to no equine-related training and that equine-handling experience alone does not guarantee the skills needed to facilitated EFL/P sessions such as equine behavior identification, “equine knowledge (including an appreciation for equine agency, and effective horse-human management” [31] (p. 14). Furthermore, many horses are retired, donated, and on second or third careers which require more physical and behavioral attention, training, and management [37]. Recent scholarship has called for practitioners to increase their equine behavior knowledge [3,36] to protect the horse’s welfare during these interactions [4]. In our vignettes, we illustrated how *critical reflexivity* might be leveraged by highly competent and educated practitioners. *Critical reflexivity* requires robust education and training in areas of equine welfare, equine behavior, human matrices of power, and instances of consent vs. choice. *Critical reflexivity* happens when the practitioner assesses anthropomorphizing behavior in session and filters it through these aforementioned areas to make informed choices during their facilitation.

However, practitioners invite anyone to join in on interactions with equids without requiring prior equine experience and encourage anthropomorphic behaviors with the equids while practitioners guide the client’s meaning-making process through a therapeutic or learning paradigm. The authors have not yet seen a conversation in the research on whether practitioners might be gatekeeping knowledge about equine behavior from clients. This could be because practitioners fear that if the client has a scientific explanation for a horse’s behavior, the EFL/P session will lose its meaning. Furthermore, does omitting behavioral information endanger equine welfare, perhaps not in the session but maybe in the long run? For example, if a client leaves a session feeling empowered by the interaction but with an incorrect interpretation of equine behavior, before applying this to equids in non EFL/P settings, who is responsible? EFL/P has a primary goal of helping humans and no broader goal of improving relationships between equids and humans. EFL/P is not presenting itself (necessarily) as a framework for horsemanship (perhaps in EAL sessions), and there is no existing research that investigates the connection of EFL/P interactions and their impact on horsemanship. However, if practitioners are committed to equine welfare, should they also not be responsible for how EFL/P interactions contribute to broader equine cultures?

We must consider the purpose and goals of EFL/P and if we are in any way (intentionally or unintentionally) aiming to understand and interpret equine behaviors and if these interpretations have any bearing on equine welfare inside and outside of sessions and beyond the lives of EFL/P equids. We argue that, for our practitioners, these interactions influence relationships beyond the session in that they carry their interpretations with them outside of EFL/P settings the data herein supported that some practitioners shared space with horses beyond the EFL/P session.

If people participate in EFL/P as a potential space to resist hierarchical horsemanship and/or to heal and learn, there is the potential to normalize anthropomorphism and perpetuate a lack of awareness of behavioral knowledge (which research has suggested impacts equine welfare) [49]. EFL/P practitioners then must ask: How can practitioners facilitate people’s experiences with horses that they perceive as positive, life-changing, and reciprocal when they are presented with a sanitized version of human–equine connection that avoids the larger welfare issues in the industry? Are we simply presenting positive experiences (what Schlote [50] calls “magic unicorn syndrome”) without requiring participants to engage in broader equine welfare that take place both inside and outside the EFL/P setting?

On a more positive note, EFL/P can be a unique space because it frontloads the work of relationship-building and reciprocity using relational and dialog work. We know that anthropomorphism, when used critically, can inspire people to act on behalf of a horse’s welfare [4] and prompt them to fight for animal welfare and influence a “public shift away from attitudes about domination and control of nature and animals” [4] (p. 11). When a practitioner uses *critical reflexivity* to work with anthropomorphism in EFL/P, there is a possibility it might elicit empathy by appealing to shared experiences among equids/humans (food insecurity and consent). It can help practitioners and clients think more relationally as well as locate shared structural inequalities that can be improved for both equid and human welfare.

This study is limited by the fact that we only interviewed practitioners who have trained or are training with the HERD Institute and not with other advanced, specialized training providers. The study only looks at anthropomorphizing from the perspective of practitioners and not of clients and participants of EFL/P. Finally, as with much of the human–equine research, a larger sample size would be advantageous towards the generalizability of findings.

## 5. Conclusions

Our vignettes used findings to illustrate how *critical reflexivity* offers choice points for well-educated practitioners in EFL/P sessions to make empowered choices with the highest level of wellbeing for both the equid and the human. Anthropomorphizing behavior can lead to welfare issues for horses in EFL/P, as we and other scholars have shown, but it can also be a key for empathizing and taking a critical and more intersectional look at the shared systems of power that affect both equids and humans.

## Data Availability

The data supporting this study’s findings are not publicly available due to privacy or ethical restrictions. The data presented in this study are available on request from the corresponding author due to privacy and ethical restrictions.
